# A haplotype of the phosphodiesterase 4D (PDE4D) gene is associated with myocardial infarction and with cardiometabolic parameters: the GEA study

**DOI:** 10.17179/excli2018-1608

**Published:** 2018-12-19

**Authors:** José Manuel Rodríguez-Pérez, Rosalinda Posadas-Sánchez, Ruben Blachman-Braun, Gilberto Vargas-Alarcón, Carlos Posadas-Romero, Esbeidy García-Flores, Fabiola López-Bautista, Carlos Alfonso Tovilla-Zárate, Thelma Beatriz González-Castro, Verónica Marusa Borgonio-Cuadra, Nonanzit Pérez-Hernández

**Affiliations:** 1 Department of Molecular Biology, Instituto Nacional de Cardiología "Ignacio Chávez", Mexico City, Mexico; 2 Department of Endocrinology, Instituto Nacional de Cardiología "Ignacio Chávez", Mexico City, Mexico; 3 Multidisciplinary Academic Division of Comalcalco, Universidad Juárez Autónoma de Tabasco, Comalcalco, Tabasco, Mexico; 4 Multidisciplinary Academic Division of Jalpa de Méndez, Universidad Juárez Autónoma de Tabasco, Jalpa de Méndez, Mexico; 5 Department of Genetics, Instituto Nacional de Rehabilitación "Luis Guillermo Ibarra Ibarra", Mexico City, Mexico

**Keywords:** phosphodiesterase, myocardial infarction, polymorphisms, Mexican population

## Abstract

The phosphodiesterase family is involved in a wide spectrum of diseases, including ischemic stroke. However, few studies have analyzed the relationship between phosphodiesterase 4D (PDE4D) and myocardial infarction (MI). Therefore, the aim of this research was to evaluate the association of the *PDE4D *gene polymorphisms with MI, and with cardiometabolic parameters in the Mexican population. Six polymorphisms (rs2910829, rs1423246, rs966221, rs4502776, rs13172481, and rs6869495) were genotyped in 1023 MI patients and 1105 healthy controls. A similar distribution of the six polymorphisms was observed in both studied groups. However, after evaluating the linkage disequilibrium, we detected a risk haplotype for MI (*AGAGAA; *OR = 1.148; P = 0.025). In addition, the polymorphisms were associated with the presence of some clinical and metabolic parameters (central obesity, hypertriglyceridemia, Aspartate transaminase >p75, Lipoprotein (a) >30 mg/dL, TAT >p75, fatty liver, and vitamin D <30 ng/dL) in healthy controls. The results suggest that in the Mexican population, a *PDE4D* haplotype is associated with increased risk of developing MI, and that *PDE4D* polymorphisms are independently associated with the presence of cardiometabolic parameters.

## Introduction

Coronary artery disease (CAD) is an inflammatory and multifactorial pathology, in which the normal function of the vascular wall and endothelium is disrupted, leading to destabilization of a coronary atherosclerotic lesion, causing rupture of the plaque and subsequent thrombus formation within the arterial lumen, that clinically presents as a myocardial infarction (MI) (Bentzon et al., 2014[4]). Currently, cardiovascular disease (CVD) represents the main cause of morbidity and mortality worldwide (Laslett et al., 2012[13]; Townsend et al., 2015[27]). Furthermore, in a recent national survey, it was shown that 25.5 % of the deaths in Mexico were attributed to cardiac and vascular pathologies, placing this as the main cause of mortality (INEGI, 2018[10]).

The underlying pathophysiology of CAD is the result of a complex interaction between different factors (i.e., smoking habit, hypertension, hyperlipidemia, diabetes mellitus, obesity, and a family history of CAD), that modified the vascular and endothelial microenvironment (Kurtoğlu Gümüşel et al., 2014[11]). Furthermore, genetic factors have been shown to have a pivotal role in the development of MI. Some genetic polymorphisms with functional implications have been associated with the development of this pathology (Asif et al., 2018[1]; Barsova et al., 2015[3]). Overall, those genetic variants are involved with molecular pathways that are essential to vascular health. In these pathways, some of the key molecules are the phosphodiesterases (PDEs), a group of enzymes implicated in the response to extracellular stimuli in the cardiovascular system. In particular, in vascular smooth muscle and endothelial cells, phosphodiesterase type 4D (PDE4D) exerts an important role through its participation in intracellular signaling pathways that modulate the concentration of cyclic adenosine monophosphate (cAMP), a secondary messenger critical to vascular function and health (Fertig and Baillie, 2018[6]; Shao et al., 2015[24]).

The *PDE4D* gene is located in the 5q12 region, and polymorphisms of this gene have been associated mainly with ischemic stroke and CAD in different populations, with controversial findings (Fidani et al., 2007[7]; Matsushita et al., 2009[15]; Milton et al., 2011[16]; Sinha et al., 2013[25]; Wang et al., 2017[29]). Thus, the aim of the present study was to investigate the association of *PDE4D* gene polymorphisms with MI in a large and well-characterized Mexican mestizo cohort, a population with characteristic genetic background (Rangel-Villalobos et al., 2008[19]; Rubi-Castellanos et al., 2009[23]; Suárez-Díaz, 2014[26]). In addition, we evaluated the association of these variants with clinical and metabolic parameters.

## Methods

### Participant selection 

In this research, participants between the ages of 30 and 75 years, belonging to the Genetics of Atherosclerosis Disease (GEA) Mexican study, were analyzed. Only unrelated and self-reported Mexican mestizo individuals (three generations at least) were included in the study (Villarreal-Molina et al., 2012[28]). A total of 1023 MI patients and 1105 controls were included. The MI diagnosis was made after a symptomatology consistent with heart ischemia.

The control group comprised participants without clinical manifestations of CAD and no familial history of premature CAD, who were enrolled through brochures posted at primary care centers (López-Bautista et al., 2018[14]). Participants with congestive heart failure, liver renal, thyroid, or oncological disease were excluded. 

In order to confirm that population stratification was not a bias or a confounding factor in the study, we previously determined a panel of 265 ancestry informative markers (AIMs) distinguishing Amerindian, European, and African ancestry. After performing this ancestry analysis (Rodríguez-Pérez et al., 2018[21]; Posadas-Sánchez et al., 2017[18]), the results showed a similar distribution in the AIMs frequencies with no statistical significance between the study groups.

The study complies with the Declaration of Helsinki and Institutional Review Board approval by the National Institute of Cardiology “Ignacio Chávez” (number 15-915). All participants approved and provided written informed consent.

### Demographic, clinical, metabolic, and anthropometric variables assessment

All participants answered standardized and validated questionnaires to obtain demographic information, nutritional habits, physical activity, smoking habit, alcohol consumption, pharmacological treatment, and familial medical history (Baecke et al., 1982[2]; Hernández-Avila et al., 1998[9]). 

As part of the GEA Study Cohort, the clinical, demographic, anthropometric, and metabolic variables, have been described in detail in our previous reports (Rodríguez-Pérez et al., 2018[21]; Posadas-Sánchez et al., 2017[17]).

### Genetic analysis 

Genomic DNA was extracted from peripherical blood samples using a conventional method (Lahiri and Numberger, 1991[12]). The polymorphisms included in the study (rs966221 (ID: C___2820039_10), rs4502776 (ID: C_____60862_10), rs13172481 (ID: C____119508_10), rs6869495 (ID: C____408428_10), rs1423246 (ID: C___1999757_10), and rs2910829 (ID: C___2820061_10) were genotyped with Taqman probes on a 7900 RT-PCR (real time - polymerase chain reaction) equipment, with manufacturer's indications (ThermoFisher, CA, USA).

### Statistical analysis

For the statistical analysis, the program Statistical Package for the Social Sciences (SPSS, version 24.0) was used. The data was analyzed according to its distribution, it was expressed as means ± standard deviation (± SD) or medians and interquartile ranges [25-75] as required, then it was analyzed with the Student t test or Mann-Whitney U as required. Categorical variables were reported as absolute frequencies and proportions and analyzed using Chi-square test. Furthermore, the Chi-square test with two degrees of freedom was used to analyze the Hardy-Weinberg equilibrium. In addition, with the aim to establish the genetic associations of the different genotypes with the disease, we performed a multivariate logistic regression analysis considering six models: additive, dominant, recessive, heterozygote, co-dominant 1, and co-dominant 2. These inheritance models have been described previously (Rodríguez-Pérez et al., 2018[21]). All models were adjusted by age, gender, body mass index, smoking habit, and type 2 diabetes mellitus. Logistic regression analysis was performed to assess the association of the polymorphic sites with metabolic parameters under different inheritance models adjusted for age, gender, and body mass index. Additionally, Bonferroni's method was made in order to considering multiple comparisons of the analyzed variables in each polymorphism. A p-value of <0.05 was considered to be statistically significant. 

Furthermore, linkage disequilibrium (LD, D´) estimations between polymorphisms and haplotype reconstruction were performed using Haploview (Broad Institute of Massachusetts, USA). 

## Results

### Study subjects

A total of 1105 controls without evidence of subclinical atherosclerosis [coronary artery calcium (CAC) score = 0] and 1023 patients with the diagnosis of MI were included in the study. Distribution of clinical, demographic, anthropometric, and metabolic parameters are shown in Table 1(Tab. 1). 

### Association and haplotype analysis of PDE4D (rs2910829, rs1423246, rs966221, rs4502776, rs13172481, and rs6869495) polymorphisms with MI

All polymorphic sites were in Hardy-Weinberg equilibrium. A similar distribution of the six polymorphisms studied was observed in both groups (data not shown). Five out of the six polymorphisms studied (rs2910829, rs4502776, rs13172481, rs6869495, and rs1423246) showed linkage disequilibrium. Ten haplotypes were constructed, and one of them associated significantly with increased risk for MI, (*AGAGAA*; OR= 1.148; P= 0.025) (Table 2(Tab. 2)).

### Association of the polymorphisms with biochemical and metabolic parameters

The effect of the *PDE4D *polymorphisms on different biochemical and metabolic parameters was explored separately in the MI patients and controls (CAC score = 0). In the MI patients, after Bonferroni's method, the data was not statistically significant (data not shown). On the other hand, in the control group, rs6869495 was associated with lower risk of central obesity (OR= 0.188, P _recessive_= 0.030; OR= 0.207, P _co-dominant 2_= 0.048), hypertriglyceridemia (OR= 0.702, P _additive_= 0.024; OR= 0.659, P _dominant_= 0.015; OR= 0.663, P _heterozygote_ = 0.024; OR= 0.656, P _co-dominant 1_= 0.021), and aspartate transaminase >p75 (OR= 0.680, P _additive_= 0.018; OR= 0.645, P _dominant_= 0.015; OR= 0.662, P _heterozygote_= 0.030; OR= 0.653, P _co-dominant 1_= 0.024). Furthermore, rs2910829 was significantly associated with lipoprotein (a) ≥30 mg/dL (OR= 0.523, P _additive_= 0.009; OR= 0.441, P _dominant_= 0.012; OR= 0.289, P _co-dominant 2_= 0.039), and rs966221 with TAT >p75 (OR= 0.513, P _recessive_= 0.032 OR= 0.475, P _co-dominant 2_= 0.028). In addition, rs1423246 was associated with lipoprotein (a) ≥ 30 mg/dL (OR= 0.413, P _additive_= 0.005; OR= 0.353, P _dominant_= 0.005; OR= 0.394, P_co-dominant 1_= 0.015), and fatty liver (OR= 0.505, P _recessive_ = 0.035). Moreover, rs4502776 was associated with vitamin D <30 ng/dL (OR= 0.701, P _additive_= 0.036). All models were adjusted for age, gender, and BMI. This data are shown in Table 3(Tab. 3).

## Discussion

In the present study, the association of six *PDE4D* polymorphisms with the risk of development of MI in the Mexican-Mestizo population was analyzed. In addition, the association of these polymorphisms with cardiometabolic parameters in controls was reported. None of the polymorphisms were independently associated with MI; however, one haplotype (*AGAGAA*) was associated with an increased risk of developing MI. In a previous study, Gretarsdottir et al. (2003[8]) performed a genome-wide association study in an Iceland population (864 patients with ischemic stroke and 908 controls), finding a strong association with some *PDE4D* gene polymorphisms (rs966221, rs12153798, rs12188950, rs702553, rs2910829, and rs1396476), and microsatellite markers. Additionally, they reported haplotypes that were associated with risk and protection for stroke; with *G0* being the most relevant haplotype, composed of SNP45 (rs1288950) and the short tandem repeat (STR) AC008818-1. Matsushita et al. (2009[15]) analyzed the same six *PDE4D* polymorphisms reported by Gretarsdottir et al. (2003[8]) in two large cohorts of patients with ischemic stroke, one cohort with 1112 patients and 1112 controls from Kyushu, Japan, and the other with 1711 patients and 1786 controls from BioBank Japan. In this study, no association with stroke was found in these Japanese populations. On the other hand, Fidani et al. (2007[7]) analyzed 97 ischemic stroke patients and 102 controls in a Greek population, and showed the same association of haplotype *G0*, that was previously reported by Gretarsdottir et al. (2003[8]).

Our results are consistent with the reports of Gretarsdottir et al. (2003[8]) and Fidani et al. (2007[7]) concerning the association of one haplotype with the risk of developing MI. In addition, our data are in line with the report by Matsushita et al. (2009[15]) because no association with the polymorphisms rs966221, and rs2910829 was detected in our patient group.

Sinha et al. (2013[25]) studied two polymorphisms (rs966221 and rs2910829) in 100 patients diagnosed with CAD and 100 controls from India. They did not directly detect association with the disease; however, they reported linkage disequilibrium (D' = 0.646) between these two polymorphisms. Additionally, they detected the association of both polymorphisms with hypertriglyceridemia. Sinha's results must be carefully analyzed, due to the D' =0.646 and the small sample size of the studied groups. 

Recently, Wang et al. (2017[29]) performed a case-control study in the Chinese Han population, analyzing four *PDE4D* polymorphisms in 610 patients with ischemic stroke and 618 controls. They established an association of rs966221 with risk for ischemic stroke; this latter finding opposes our results. The risk analysis of *PDE4D *polymorphisms with different components of the CVD has shown different associations according to the ethnicity of the studied groups. Thus, the genetic heterogeneity between populations might be responsible for the controversial results reported in the current literature (Rosand et al., 2006[22]). It is important to consider that that the majority of previous studies of *PDE4D* polymorphisms were performed in Caucasian or Asian populations; thus, those results do not always provide information that applies to the Mexican population.

On the other hand, when we analyzed both groups independently (MI patients and controls) with just the control group, five polymorphisms were associated with some metabolic parameters and cardiovascular risk factors. Currently, few studies of *PDE4D* have analyzed the association between the genotype and risk factors related to MI. 

In this sense, Gretarsdottir et al. (2003[8]) evaluated the association of alleles and haplotype with factors classically associated with stroke (i.e., CAD, peripheral artery occlusive disease, diabetes mellitus, hypercholesterolemia, and hypertension), and the authors did not find any risk factor associations. Contradictory, those findings were not consistent with the results of an Indian population with CAD, in which two polymorphisms (rs966221, and rs2910829) were associated with hypertriglyceridemia (Sinha et al., 2013[25]). Regarding this, we detected various associations related to lower risk in the control group, suggesting that these polymorphisms may confer protection against the presence of these metabolic abnormalities and cardiovascular risk factors. 

Another important point to consider is that MI includes complex mechanisms that involve not only changes at the DNA level, but also at the epigenetic, post-transcriptional, or post-translational level, or may involve alternative splicing modifications (Duan et al., 2018[5]; Rodríguez-Pérez et al., 2016[20]).

Some limitations should also be considered, which include the inherent limitations of a cross-sectional study and the inherent survival bias in which only MI patients that survive and arrived to the hospital were analyzed. However, we consider that the present study has important strengths which include a large cohort of Mexican individuals with and without MI; we have a highly superior number of patients compared to previous studies (Fidani et al., 2007[7]; Sinha et al., 2013[25]; Wang et al., 2017[29]). For the control group, only subjects with CAC score = 0, as evaluated through a computed tomography, were analyzed; thus, in the control group there were no cases of participants with subclinical atherosclerosis. Population stratification was not biased as an important confounder factor, as the proportions of Caucasian, Native American and African ancestries were similar in both study groups. To the best of our knowledge, none of the polymorphisms studied had been previously analyzed together to assess the risk of *PDE4D* gene with MI.

## Conclusion

In summary, our results suggest that none of the analyzed polymorphisms of *PDE4D* were independently associated with MI. However, one haplotype was associated with risk (*AGAGAA*) for MI. Thus supporting the role of haplotype susceptibility as a marker for MI in our population. Furthermore, it was shown that different polymorphisms of this gene were associated with metabolic parameters and cardiovascular risk factors in the Mexican population.

## Acknowledgements

The authors are grateful to the study participants. Moreover, these results were part of Ruben Blachman's research project during his Master's studies. This study was supported by grants from the Nacional Council for Science and Technology (CONACYT project number: 233402).

## Disclosure

All researchers declare that there were no conflicts of interest related to this article. 

## Figures and Tables

**Table 1:**
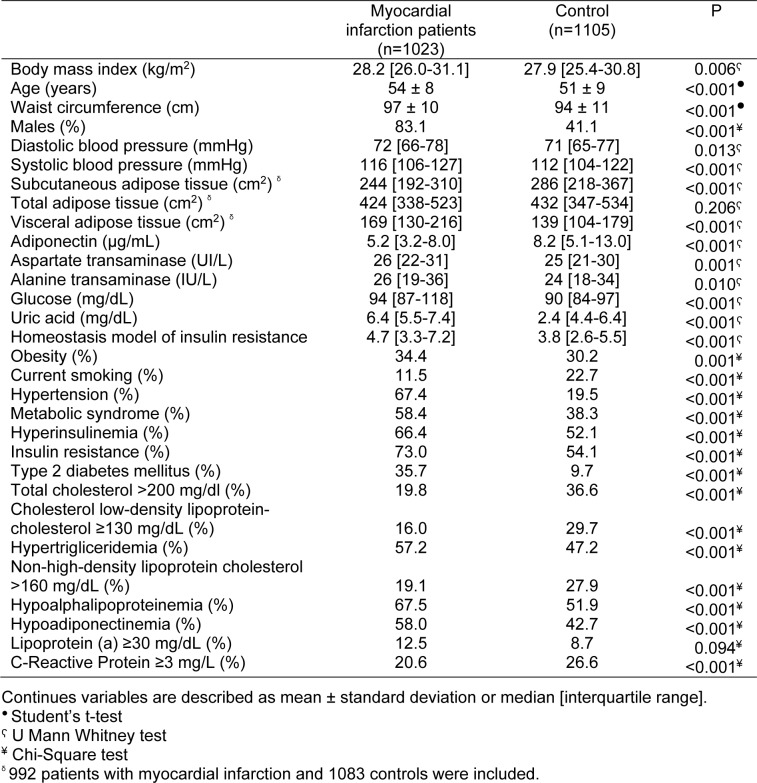
Demographic, clinical, and biochemical variables in the studied groups

**Table 2:**
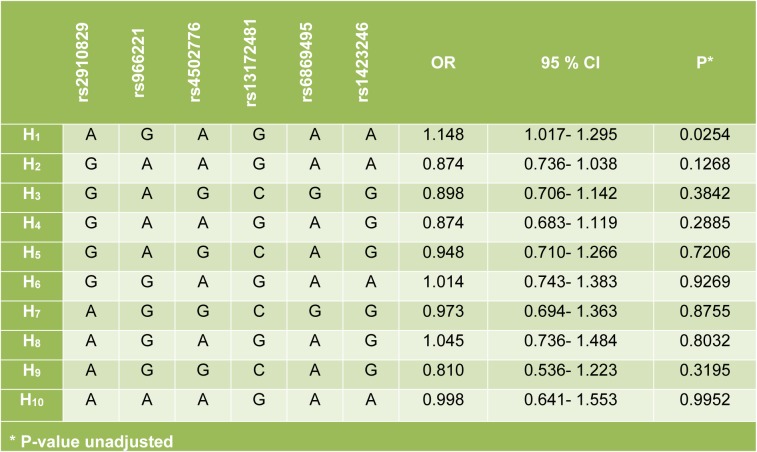
Haplotype analysis of the *PDE4D* gene polymorphisms

**Table 3:**
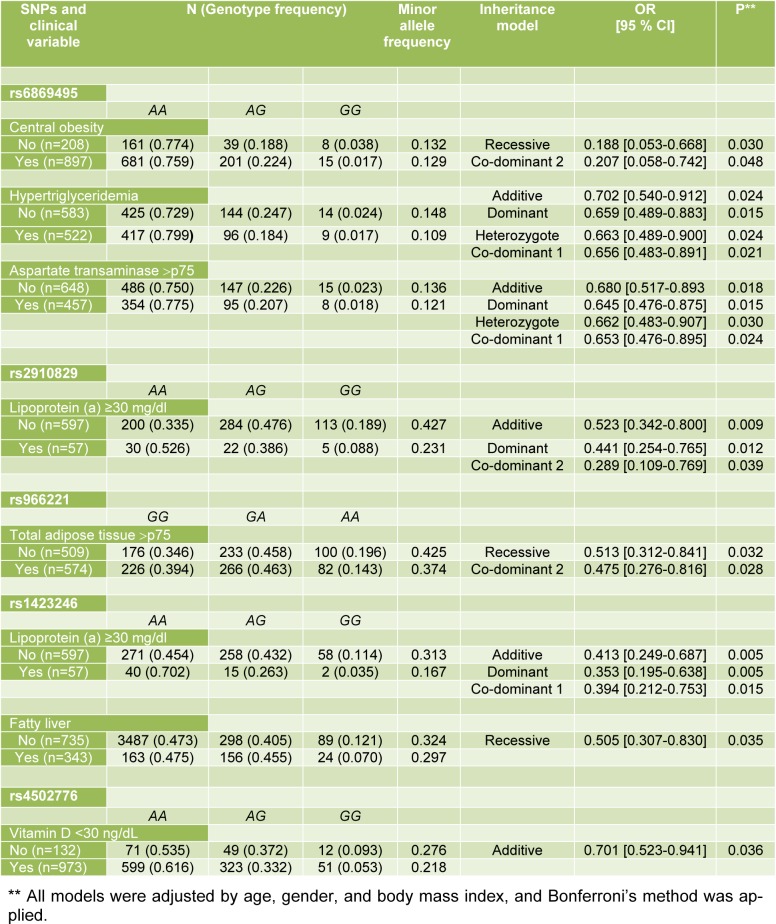
Association of the *PDE4D* polymorphisms with clinical and metabolic anomalies in the control group
